# Polarization-insensitive planar patch antenna with large embedded serial capacitance for on-metal tag design

**DOI:** 10.1038/s41598-023-34836-y

**Published:** 2023-05-11

**Authors:** Muthukannan Murugesh, Eng-Hock Lim, Pei-Song Chee, Fwee-Leong Bong

**Affiliations:** 1 Department of Electrical and Electronic Engineering, Universiti Tunku Abdul Rahman, 43000 Kajang, Malaysia; 2 Department of Mechatronics and Biomedical Engineering, Universiti Tunku Abdul Rahman, 43000 Kajang, Malaysia; 3 HID Global, 81000 Skudai, Malaysia

**Keywords:** Electrical and electronic engineering, Physics

## Abstract

A polarization-insensitive planar patch antenna, which has a large embedded serial capacitance, is proposed for constructing a metal mountable tag by using merely a single radiator. The proposed antenna structure itself contains two flaps of patches, which are very closely overlapped, for generating a large capacitive reactance for reducing the resonant frequency of the tag. It has been found that the surface currents in the overlapped region are in the reverse direction as the large capacitance is virtually placed in series. This feature has been tactfully employed for producing a pair of orthogonal currents for designing the polarization-insensitive tag antenna and it can generate orthogonal fields in a unique way, making it readable from almost all directions at all points above the metal surface. For analyzing the impedance properties, an equivalent circuit was also constructed. This tag antenna is compact, and it can be read from ~ 15 m with 4W EIRP. Furthermore, the tag resonant frequency is shown to be unaffected much by its backing material. The proposed tag antenna is polarization-insensitve as it can be accessible from almost all directions on the metal.

## Introduction

In the last few years, radio frequency identification (RFID) technology has been used in many practical applications such as electronic toll collection, retail management, inventory tracking, and remote access. The new technology uses radio waves to identify and track objects automatically^1^. This is because RFID has a higher reading range, faster reading capability, and more memory size than the ordinary barcodes. An embedded microchip in a passive UHF RFID tag stores an exclusive object code that can be used to identify an attached microchip can be energized by the radio waves from the reader antenna. However, the UHF RFID detection method has some physical limitations. First, when a conventional UHF tag is put on a metal surface, its reading range can be significantly reduced^2^. To overcome this problem, a couple of antenna structures such as folded patches^3^, inverted antennas^4^, and grounded dipoles^5^ have been utilized for constructing metal-mountable tags.

Another limitation is that most of the tags in the market are only readable in a certain polarization. This may result in a considerable rise in the miss rate. In the past decade, many linearly polarized (LP) antennas have been used to design metal tags. They include dipoles with artificial magnetic conductor (AMC) structure^6^, microstrip patch antenna^7^, and planar inverted-F antenna^8^. The LP tag is usually read using a circularly polarized (CP) reader antenna, regardless of the tag’s orientation. However, as mentioned in^9^, there is always a 3-dB polarization mismatch between the LP tag and the CP reader antenna, which reduces the detection distance. Several CP tag antennas have been proposed in recent years to address this issue, but only a few^10–12^ have been found to be suitable for usage on metal. However, their footprints (> 70 mm × 70 mm) are simply too huge for a small object. In addition, it is usually difficult to optimize a CP metal tag as the effects of the backing metal on the axial ratio must be carefully considered. A tagged item can be placed arbitrarily in many practical applications, such as wrapping it around a piece of cardboard or hanging it on a rack. The tag is required to read effectively in all scenarios^13^. Because of this, different types of linearly polarized tag antennas with orientation insensitivity have been investigated. Wrapping two dipoles around 3D structures like spheres and cubes can enable accessibility in all directions^13,14^. However, the 3D structures have a large profile and are difficult to fabricate.

The demand of planar polarization-insensitive UHF tags has been growing as such radiators can provide readability in all polarizations. Combining multiple single-polarized resonators is definitely a possible way to design an antenna that can read in all polarizations. In^9^, two dual-planar inverted-F antennas are placed in cross arrangement and fed through two balanced ports to provide polarization insensitivity in all directions. However, the tag antenna is large (64 mm × 64 mm), and it requires the use of four lumped capacitors and 12 vias. This can also introduce uncertainty as some antennas are very sensitive to the locations of vias. A polarization-insensitive UHF tag antenna that can be mounted on metal surface has been proposed^15^, where two dipolar patches are positioned orthogonally for designing a tag that can be detected in all directions in the upper space (*θ* ≤ 90°). The blind spots of either dipole have been effectively removed by combining the fields generated by the other. However, the reported tags in^9^ and^15^, both of which have employed two pair of radiators, have a short read range (less than 3.5 m), and they require a four-port microchip (Monza 4D). The concept of integrating an inductive channel with a lumped external inductor was presented in^16^ for adjusting the resonant frequency of a bowtie dipolar tag arbitrarily. It was shown that the currents that flowed on the top-loading patch could be easily regulated by the inductive channel, and the resonant frequency of the tag antenna could be effectively adjusted by changing the channel width as well as the external lumped inductor. However, the tag antenna has a high profile with a two-layer design, and it cannot be read in all polarizations. Two complementarily placed C-shaped patches have been used to design a metal-mountable tag with a wide range of frequency tuning capabilities^17^. To generate sufficient antenna reactance, the two patches are closely coupled. This reactance is then employed as a tuning mechanism and effectively tuned over a wide range. Nevertheless, the tag antenna has a high profile, and it cannot be read in all polarizations.

For the first time, a new planar patch antenna, which has a large embedded capacitance, is employed for constructing a linearly polarized polarization-insensitive tag antenna, which can be used for metal-mountable applications. Many methods have been explored in the past decades for generating capacitive effect in the microstrip antennas for achieving different purposes. It was found in the late 70s that the capacitive coupling of the overlapped microstrip dipole antennas could be employed for increasing the antenna bandwidth and efficiency^18^. Different capacitively loaded microstrip ring antennas were proposed for achieving a smaller size, improving the impedance matching, and attaining a higher Q factor^19,20^ in the past years. It was found that capacitive loading can be introduced to a microstrip patch antenna by stacking the patches^21,22^. The concept was also applied for designing a linearly polarized UHF RFID tag antenna in^23^, where it was found that stacking multiple patches can generate sufficient capacitance so that the antenna impedance can achieve good impedance matching with the microchip, with the price of increasing the tag profile significantly. Different from the conventional stacking method, our newly proposed tag antenna has employed the capacitive coupling mechanism to generate orthogonal currents for designing a polarization-insensitive tag antenna for the first time. The proposed tag design has two flaps of patches that are closely overlapped for generating sufficient capacitive reactance to reduce the tag antenna’s resonant frequency to the UHF RFID frequency range. Also, the surface currents on the overlapped region are found to be in the opposite direction as those on the non-overlapped portions. Here, the antenna structure is tactfully modified so that the currents in the overlapped patch region can be made orthogonal with those in the non-overlapped ones. The pair of the orthogonal currents are then employed for designing a polarization-insensitive tag antenna using a single piece of radiator. This method is novel because it can be used to design a polarization-insensitive tag antenna without the need of multiple radiating components. To our best knowledge, we are the first to apply such technique to design a polarization-insensitive tag antenna. Since the two flaps of patches are closely overlapped, it does not increase the antenna profile. It should be mentioned that it is usually very challenging to design a UHF polarization-insensitive tag antenna that can be used on metal surfaces. We also proposed the first polarization-insensitive tag antenna in^15^, but it would require the use of two pairs of dipolar patches and a four-port chip. On the other hand, the newly proposed tag antenna structure only needs a two-port chip. No metallic vias are required here since the antenna layout can be etched on a copper laminated inlay using the regular Printed Circuit Board (PCB) etching. Since the presented antenna includes a ground plane to separate the radiator from the backing metal, it can be applied for designing a polarization-insensitive tag that is useable on metal.

## Methods

### Tag antenna configuration

**Figure 1:**
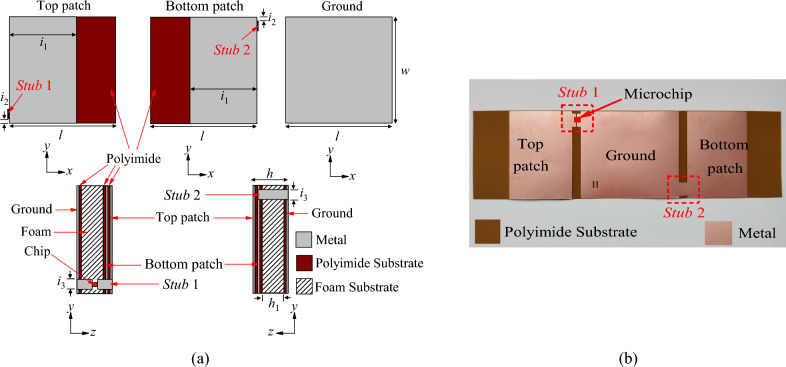
(**a**) Configuration of the proposed tag antenna (*l* = 40 mm, *w* = 40 mm,* h* = 3.38 mm, *h*_1_ = 3.2 mm, *i*_1_ = 25 mm, *i*_2_ = 1.1 mm, and *i*_3_ = 6 mm) and (**b**) Flexible antenna inlay.

**Figure 2:**
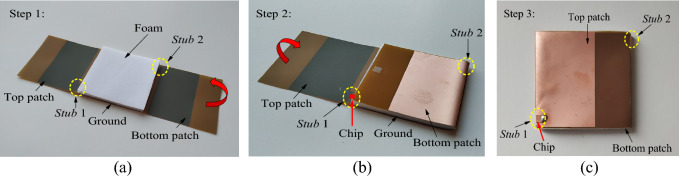
Fabrication process of the tag antenna by folding. (**a**) Step 1: A foam with square footprint is attached on the ground patch. (**b**) Step 2: The bottom patch is folded so that it can cover the foam substrate. (**c**) Step 3: The top patch is folded overlapping with the bottom patch for forming the final tag structure.

Figure 1a shows the configuration of the proposed tag antenna. The antenna inlay contains two patches (*w* × *i*_1_) as well as a ground, both of which are made on a thin layer of copper (thickness of 0.009 mm) located on a flexible thin polyimide substrate with a thickness of 0.05 mm, as shown in Fig. 1b. The inlay is simple to make using the regular PCB fabrication techniques. The tag antenna’s footprint was first reproduced on a photoresist mask that had already been laminated on the inlay's copper layer. The unwanted copper was then removed by etchant after being exposed to ultraviolet light. Through two metallic stubs, which are named as *Stub* 1 and *Stub* 2, the top and bottom patches are joined to the ground, and an integrated RFID microchip is bonded across the gap at the center of *Stub* 1. A Ucode 8 ^24^ microchip, with an actual read sensitivity of − 20.85 dBm and an input impedance of 13 − *j*191 Ω at 915 MHz, is used for the tag design. Next, the tag is formed by folding the inlay. The folding technique is now elaborated, as shown in Figs. 2a–c. First, a polyethylene foam substrate (*ε*_*r*_ = 1.03, tan *δ *∼ 0.0001), with geometrical parameters of (*w* = *l* = 40 mm and *h*_1_ = 3.2 mm), is placed on the ground. The flap that contains the bottom patch is then folded wrapping around the foam, as depicted in Fig. 2a, using adhesive. Next, the top patch is folded overlapping with the bottom patch, as shown in Fig. 2b, forming the final tag structure in Fig. 2c. It should be mentioned that the microchip is soldered across the gap at the center of *Stub* 1. As can be seen in Figs. 1 and 2, the top and bottom patches are closely stacked and their metallic layers are partially overlapped, where the two patches are only separated by a thin layer of polyimide substrate. The overlapped patches can introduce sufficiently large capacitance that is crucial for bringing down the resonant frequency to the useful UHF RFID band. It will be later shown that the overlapped region is also important for introducing reverse surface currents that are instrumental for the generation of the polarization-insensitive radiation pattern. Here, the bottom and top patches are connected to the ground through *Stub* 1 and *Stub* 2, which are now located on the vertical sidewalls after the folding process, in the final tag structure. In this case, the ground patch acts as an isolator for reducing the effect of the backing item. The chip goes to the vertical wall as well, avoiding from being easily hit by an external object. The foam material is soft, and it can provide a certain level of flexibility. As a result, the tag can be attached to an object with a slightly uneven curvature. Additionally, the foam functions as a structural support, ensuring that the tag can hold its shape. This tag is simulated by attaching its ground at the middle of a 20 cm × 20 cm metal plate. The simulation software (CST Studio Suite) has been used for all the optimization and design processes.

### Fabrication process

**Figure 3:**
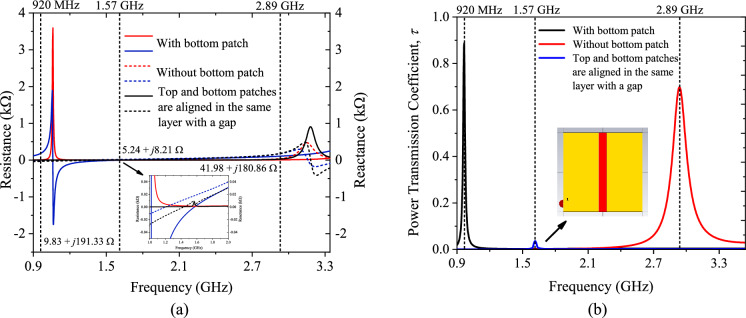
The tag configuration for the cases with and without the bottom patch and both patches are aligned in the same layer with a gap (**a**) impedance analysis and (**b**) power transmission coefficient analysis.

To begin the simulation, the microchip is replaced by an external discrete port with a complex impedance set to represent the actual chip impedance (13 − *j*191 Ω). The antenna design is initially analyzed by removing the bottom patch as well as *Stub* 2 for establishing a single-layer tag. As shown in Fig. 3a, the tag antenna's resonant frequency has moved up to 2.89 GHz with an antenna impedance of (41.98 + *j*180.86 Ω), which goes far beyond the regulated UHF RFID band due to a significant reduction in reactance. Although the realized gain is high (6.601 dBi), the power transmission coefficient is low (0.67), as shown in Fig. 3b, due to poor impedance matching. Next, the top and bottom patches are aligned in the same layer with a gap between them. The tag antenna's resonant frequency has reduced to 1.57 GHz with an antenna impedance of (5.24 + *j*8.21 Ω). As a result, the realized gain is very low (− 13.85 dBi), and the power transmission coefficient has also become very low (0.05) due to poor impedance matching. After including the bottom patch in separate layer, the frequency can be shifted back to 920 MHz. It shows that the existence of the overlapped region, which will be further discussed in detail later, is essential for reducing the tag resonant frequency to the 860 MHz–960 MHz frequency range. The final tag antenna’s impedance and realized gain are 9.83 + *j*191.33 Ω and 1.07 dBi, respectively, as shown in Fig. 3a, which has achieved an excellent power transmission coefficient of 0.9.

### Design analysis and working principle

**Figure 4:**
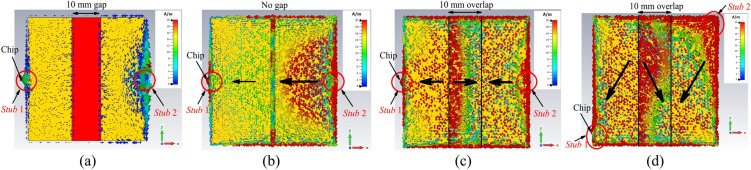
Surface currents on the top and bottom patches for the tag antenna with (**a**) 10 mm gap (**b**) no gap (**c**) 10 mm overlap and (**d**) 10 mm overlap with shorting stubs moved to the corners.

The surface currents on the top and bottom patches are analyzed next to demonstrate the working concept of the tag. Design simulation is begun by varying the patch length *i*_1_, where the two shorting stubs are both shifted to the center. First, the patch length is shortened to *i*_1_ = 15 mm so that there is a gap of 10 mm left in between the two patches, as shown in Fig. 4a. Here, the tag resonant frequency has increased to 2.0456 GHz, with a poor reflection coefficient of |S_11_| = − 0.0259 dB. The two patches are not well excited at this frequency and weak surface currents are observed due to weak capacitive coupling. Subsequently, the two patches are made equal in length (*i*_1_ = 20 mm), as shown in Fig. 4b, where each has one edge aligned along the centerline, resulting in no gap. Due to the tight capacitive coupling between the edges, the tag resonance has gone down to 1.4207 GHz and the reflection coefficient has improved to − 3.252 dB. Here, the formation of a completed circuit loop has enabled stronger currents to flow in one direction. The patch lengths are then increased to *i*_1_ = 25 mm, resulting in an overlapped region of 10 mm, as depicted in Fig. 4c, again, with the two shorting stubs at the center. Strong capacitive coupling is made possible due to close proximity between the two patches in the overlapped region. As previously mentioned, the separation distance between the top and bottom patches is very thin and it is only about the thickness of a polyimide substrate. Also, the surface currents in the overlapped region are found to be in the opposite direction as those in the non-overlapped regions. Because of the strong capacitance, the tag resonant frequency has been reduced to 1.0818 GHz. Enhancement in the capacitive reactance has also improved the reflection coefficient to − 18.03 dB. Finally, with reference to Fig. 4d, the two shorting stubs are symmetrically displaced (for the case of 10 mm overlap), with *Stub* 1 moved to the lower left and *Stub* 2 shifted to the upper right, and the corresponding currents are simulated. The displacement has caused the current path to become longer and the tag resonant frequency to shift down to 920.3 MHz (with |S_11_| = − 14.21 dB).

**Figure 5:**
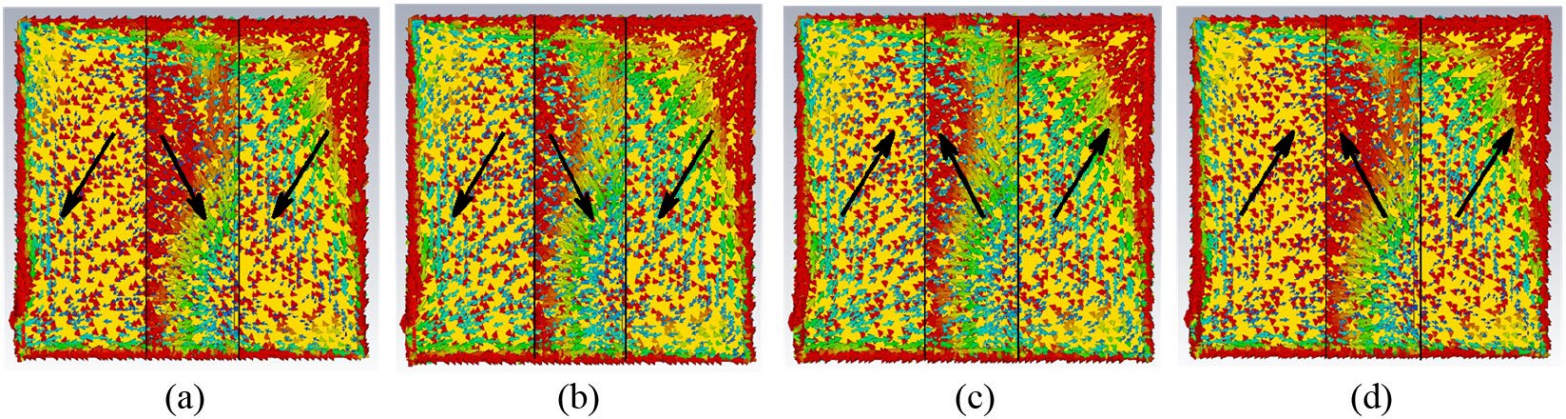
Surface currents on the top and bottom patches at the source phases of (**a**) 0° (**b**) 90° (**c**) 180° and (**d**) 270°.

It also causes the currents in the overlapped region to become orthogonal with those in the non-overlapped regions. The pair of orthogonally oriented currents has enabled the formation of the polarization-insensitive radiation pattern. The surface currents are also captured at different snapshots for the source phases of 0°, 90°, 180°, and 270°, as illustrated in Figs. 5a–d. The current distributions on the radiator patches are in-phase at the source phase of 0° and 90° as their source currents are flowing in the same direction. The current distribution at the source phase 180° becomes out-of-phase due to the change of current direction. Two flows of linearly polarized diagonal currents, which are mutually orthogonal, are observed in the overlapped region. Each of the currents can be decomposed into its subcomponents in the *x*- and *y*-directions. The two diagonal currents are instrumental for generating the *E*_*θ*_ and *E*_*ϕ*_ fields, which are orthogonal in nature, for the polarization-insensitive tag antenna. At each snapshot, the orthogonality of the currents can be maintained. This has ensured the sustainability of polarization insensitivity in the radiation pattern when time elapses.

### Equivalent circuit and parametric analysis

**Figure 6:**
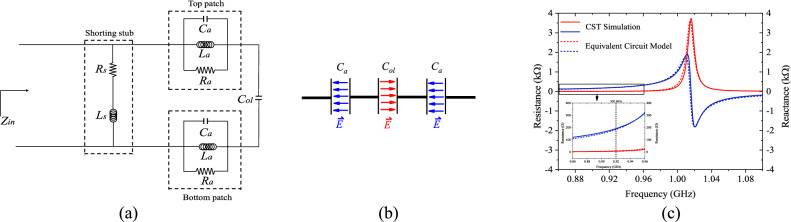
(**a**) Proposed tag antenna’s equivalent circuit model (*R*_*s*_ = 5.21 mΩ,* L*_*s*_ = 1.58 nH,* R*_*a*_ = 1.84 kΩ, *L*_*a*_ = 8.23 nH, *C*_*a*_ = 1.54 pF, *C*_*ol*_ = 70.83 pF). (**b**) Fields in the capacitors in series. (**c**) Comparing the modeled and simulated input impedances of the proposed tag antenna.

Figure 6a illustrates the tag antenna's equivalent circuit. It is set up here for the purpose of analyzing the antenna’s characteristics. The top and bottom patches are equal in dimension, and both are joined to the ground by a shorting stub, where each patch can be described by the lumped components *R*_*a*_, *L*_*a*_, and *C*_*a*_ arranged in parallel. The total patch impedance is *Z*_*a*_ = *R*_*a*_||*L*_*a*_||*C*_*a*_. The top and bottom patch inductances *L*_*a*_ can be obtained using *L*_*a*_ = 200*i*_1_{ln [2*i*_1_/(0.5(*w* + *w*) + *C*_*t*_)] + 0.50049 + [(0.5(*w* + *w*) + *C*_*t*_)/3*i*_1_]} (nH)^25^. The capacitance can be calculated using the equation *C*_*a*_ = *ε*_*r*_*ε*_*o*_*A*_*e*_/*h*_1_ (pF) ^26^, here, *h*_1_ and *ε*_*r*_ are the foam substrate’s thickness and relative permittivity, respectively. Taking electric fields into account, the space between the patches and the ground patch is also seen as parallel capacitive plates, where *A*_*e*_ = *i*_1_(*w* + *w*)/2 is the patch’s effective area. A macro model method^27^ can be used to determine the resistance *R*_*a*_. Shorting stubs are modeled here by a serial connection of a resistance *R*_*s*_ and an inductance *L*_*s*_. The shorting stubs have a resistance of *R*_*s*_ = 2[(*ρh*)/(*i*_3_ × *h*_1_)][*K*_*c*_/(1 − e^−*x*^)]^28^, in which the copper’s resistivity is *ρ* = 1.72 × 10^−8^ Ωm, The current crowding factor is *K*_*c*_ = 1.94, and *x* = [2(1 + *h*_1_/*i*_3_)] (*δ* /*h*_1_), where *δ* = 2.18 × 10^−6^ m is the copper skin depth. *L*_*s*_ = 400*h*{ln [2*h*(*C*_*t*_ + *i*_3_)] + 0.50049 + [(*C*_*t*_ + *i*_3_)/3*h*]} can be used to calculate the inductance of the shorting stub. The overlapped region of the top and bottom patches has introduced an additional capacitance and it can be calculated by using *C*_*ol*_ = *ε*_*r*_*ε*_*o*_*A*/*d*, where *A* is the overlapped area and *d* is the separation distance. A large capacitance has been resulted as *d* is as thin as the polyimide thickness. As can be seen from the equivalent circuit in Fig. 6a, the two *RLC* resonators are serially connected to *C*_*ol*_. Since *C*_*ol*_ is serially cascaded to the two *C*_*a*_, forming *C*_*a*_ - *C*_*ol*_ - *C*_*a*_ virtually, as can be observed in Fig. 6b, the electric fields in the overlapped region must be in the opposite direction as those in the non-overlapped regions, resulting in opposite surface currents as well. All the calculated element values are shown in the figure caption. The proposed tag antenna’s total input impedance (*Z*_*in*_) can then be described using Eq. (1). The equivalent circuit model helps to determine the antenna’s impedance. For tag design, it is always an objective to make *Z*_*in*_ = Z*_chip_ so that conjugate matching condition can be attained in between the antenna and the chip, enabling a maximum power transfer. The equivalent circuit model’s input impedance is then shown in Fig. 6c and matches well to that obtained by the simulation software. There is a slight difference between the curves since the component values are calculated by assuming uniform current distribution on the radiating patches. Regardless of the slight difference, the equivalent circuit can still be useful for impedance analysis. Since the tag antenna’s port is aligned along the sidewall of the flexible foam substrate, reaching it with a differential probe for antenna impedance measurement is quite difficult. So, this section only includes the simulated antenna impedance.

1 $$Z_{in} = \left( {2Z_{a} + \frac{1}{{j\omega C_{ol} }}} \right) || Z_{s}$$

where

2 $$Z_{a} = \frac{{j\omega R_{a} L_{a} }}{{R_{a} - \omega^{2} R_{a} L_{a} C_{a} + j\omega L_{a} }}$$

3 $$Z_{s} = R_{s} + j\omega L_{s}$$

**Figure 7:**
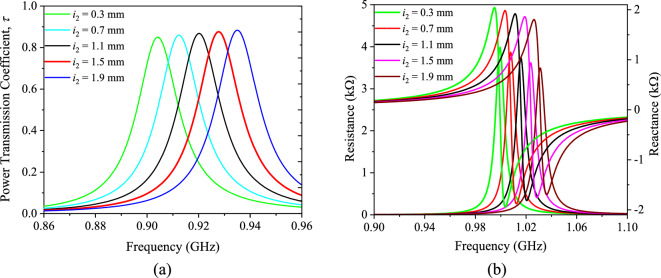
Analyzing the (**a**) power transmission coefficient and (**b**) impedance by varying the offset distance (*i*_2_) of the shorting stubs.

**Figure 8:**
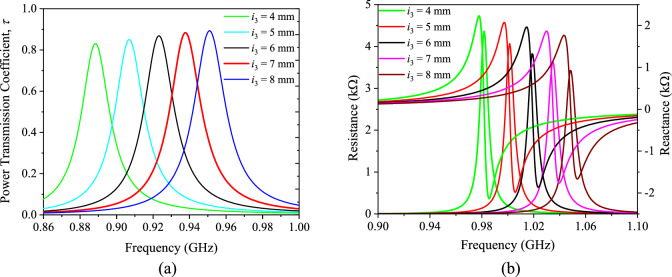
Analyzing the (**a**) power transmission coefficient and (**b**) impedance by changing the width of the shorting stubs *i*_3_.

Simulations were used to investigate the impact of the key design parameters on the power transmission coefficient (*τ*) and input impedance. The tag is always attached to the middle of a 20 cm × 20 cm metal plate. The offset distance (*i*_2_) of the shorting stubs is studied first. Here, *i*_2_ is increased from 0.3 mm to 1.9 mm to perform frequency tuning. As seen in Fig. 7a, decreasing *i*_2_ contributes to lowering the tag resonant frequency but keeping the *τ* value almost a constant. The resonant frequency of the tag is seen to be decreasing at a rate of 17.5 MHz with increasing *i*_2_ by a step size of 0.4 mm. As the stub’s offset is reduced, the tag antenna becomes more resistive (tag resistance raises) and inductive (tag reactance raises) at the tag resonant frequency, as observed in Fig. 7b. Adjusting the offset location is therefore important for rough tuning the tag antenna’s resonant frequency to the appropriate UHF RFID passband. The effects of the shorting stubs width (*i*_3_) are then studied. As shown in Fig. 8a, raising *i*_3_ from 4 to 8 mm makes the tag antenna’s resonant frequency moving higher, but it retains a constant *τ*. When the width of both the shorting stubs is decreased from 8 to 4 mm, the tag’s resistance and reactance stay almost unchanged, even though the corresponding resonant frequency has dropped down.

## Results

**Figure 9:**
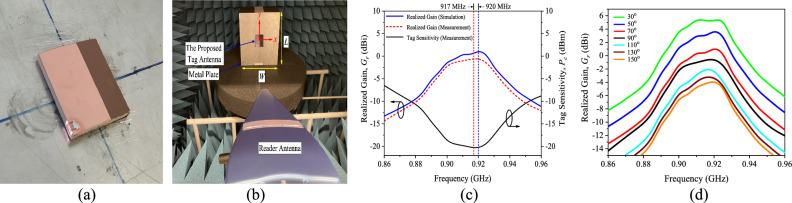
(**a**) Aluminium plate with the prototype attached at the middle. (**b**) The measurement setup in the anechoic cabin. (**c**) Simulated and measured realized gains, as well as the measured tag sensitivity, when the tag is placed on a 20 cm × 20 cm aluminium plate and (**d**) Measured realized gains at different locations.

The performance of the tag inside an anechoic cabin was tested using the Voyantic Tagformance Measurement System^29^. With reference to Fig. 9a, the tag is attached to the center of a 200 mm × 200 mm aluminium plate, which is kept in place by a Styrofoam with a relative permittivity of 1, in all measurements. The tag is always placed at the center of the cabin. The fabricated prototype and the measurement setup are depicted in Figs. 9a and b, respectively. The proposed tag is tested using an LP antenna reader (8 dBi Gain). The distance between the reader antenna and the tag being measured along the *z* axis is kept constant at 52 cm. The realized gain is defined as *G*_*r*_ = *P*_*ic*_/(*L*_*T*_ × *P*_*tx*_), where *L*_*T*_ is the total cable and free space losses, *P*_*ic*_ is the read sensitivity of the microchip (–20.85 dBm), and *P*_*tx*_ is the reader’s threshold power level at a specific frequency. Figure 9c depicts our tag antenna’s simulated and measured realized gains. The maximum realized gain at 917 MHz is measured to be –1.12 dBi, with reasonable agreement observed between the simulation and measurement results. Figure 9d shows the proposed tag antenna’s measured realized gains at different locations in the range 30° ≤ *θ* ≤ 150°. The maximum realized gain at *θ* = 30° is measured to be 5.7 dBi.

**Figure 10:**
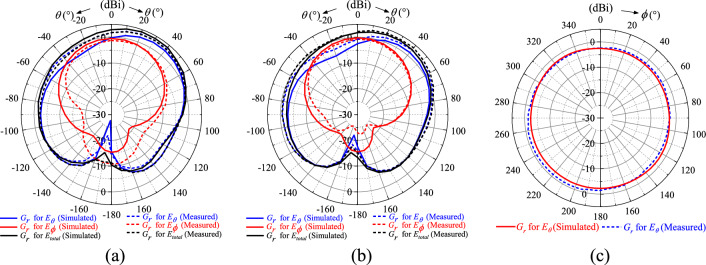
Simulated and measured realized gains in the (**a**) *xz* plane (**b**) *yz* plane and (**c**) *xy* plane.

**Figure 11:**
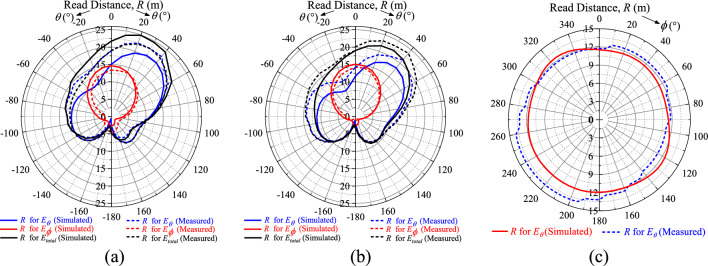
Simulated and measured read distances in the (**a**) *xz* plane (**b**) *yz* plane and (**c**) *xy* plane.

Then, the simulated and measured realized gains (*G*_*r*_) of the polarization-insensitive tag antenna are studied in the *xz*, *yz*, and *xy* planes, as shown in the polar plots in Figs. 10a–c, depicting the realized gains for the fields *E*_*θ*_, *E*_*ϕ*_, and *E*_*total*_ = $\left| {\mathop{E}\limits^{\rightharpoonup} _{total} } \right| = \sqrt {\left| {\mathop{E}\limits^{\rightharpoonup}\theta } \right|^{2} + \left| {\mathop{E}\limits^{\rightharpoonup} \phi} \right|^{2} }$. The simulated and measured results are in good agreement. The *E*_*ϕ*_ component diminishes when it approaches *θ* = 90° as tangential electric field does not exist on metal. Here, only the *E*_*θ*_ component, which is normal to the metal surface, can exist. For all cases, the field maximizes at around *θ* = 40° due to the presence of the two shorting stubs in the diagonal corners, where the surface currents are the strongest. Since the tag is mounted on the metal plate, the region of interest is the upper hemispherical space (*θ* ≤ 90°). As seen in Figs. 10a and b, the realized gains are reasonably good for both the *E*_*θ*_ and *E*_*ϕ*_ components in the upper hemisphere, signifying that the tag can be read in both the *θ* and *ϕ* directions above the metal plate. The *G*_*r*_ for *E*_*total*_ is larger than 0 dBi for (*θ* ≤ 50°), and it shows polarization insensitivity feature in the *xz* and *yz* planes, with a nonuniformity factor of less than 4 dB in the entire region (*θ* ≤ 90°). As can be seen from Fig. 10c, the field pattern is nearly uniform in the *xy* plane (*θ* = 90°), where the difference between the maximum and minimum points is less than 2 dB. In this plane, the maximum realized gain is achievable in the angular range of 195°–260° and it is slightly skewed as stronger fields are generated in the directions of 45° and 225°, which are the locations of the shorting stubs, as can also be observed in Fig. 5, where stronger currents are induced around the shorting stubs. To have a better view on the read performances when the tag is placed on metal, the corresponding measured and simulated read distances (*R*) have been added as Fig. 11. The tag can be read reasonably well in the upper-half space (*θ* < 90°) in both the azimuthal and elevation directions. In conclusion, the suggested tag antenna can be read in all directions at all points above the metal plate.

## Discussion

**Figure 12:**
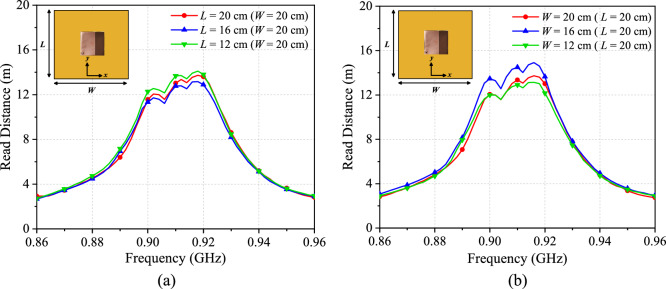
The tag antenna’s measured read distances on a metal plate by varying the (**a**) length of plate *L* and (**b**) width of plate *W*.

The tag antenna is suitable for use on metallic surfaces. It is now tested on aluminium plates that are cut into different dimensions (*W* × *L*), all of which have a thickness of ~ 5 mm. The prototype was always attached to the middle of the plate during the tests. For comparison, maximum read distances were measured in the boresight (*θ* = 0°) direction. When *W* is kept at 20 cm, the read range can be maintained ~ 13 m by lowering *L* from 20 cm to 12 cm progressively, as shown in Fig. 12a. The plate width *W* is then varied while the plate length *L* remains constant at 20 cm, and the results are depicted in Fig. 12b. It was observed from simulations that the read ranges fluctuate within a very narrow range of 14.71 m–14.74 m when the plate dimension is varied. We have concluded that the discrepancy can be caused by experimental tolerances such as the placement accuracy and others. When the plate width is reduced, the read distance drops. Worth to mention here is that the tag resonant frequency is stable for all cases.

**Figure 13:**
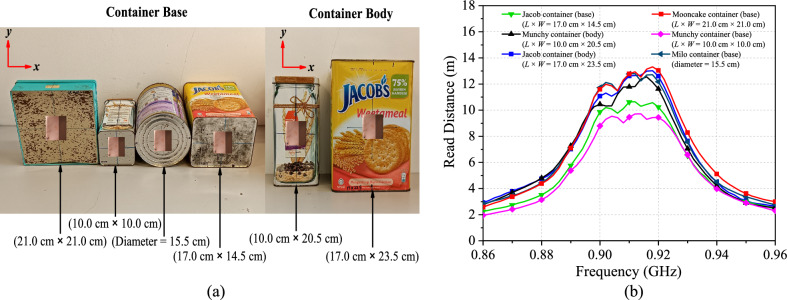
(**a**) Different metallic household items and (**b**) Read distances for the different metallic household items.

The tag was also tested using a couple of metallic household items for verifying its practical implementation. With reference to Fig. 13a, the tag was placed on the body and the base of the household items to acquire their read ranges in the boresight (*θ* = 0°) direction. The measurement curves are shown in Fig. 13b. When the tag is attached to the household items, a far read range of 10 m–13 m is achievable. The read distance does decrease with decreasing the surface. Even though the metallic containers vary in material and size, the tag resonant frequency remains consistent. The tag resonant frequency can be controlled within a small range of 917 MHz–919 MHz, while the reading performance remains stable. It has been proven that our tag antenna is less sensitive to the backing metallic object, which can become a useful feature for practical applications.

Comparison is made between the proposed tag antenna with other on-/in-metal tag antennas, as tabulated in Table 1. Two dual-planar inverted-F antennas are placed in cross constellation for designing a polarization-diversity antenna that can be used for metal-mountable applications^9^. But the tag size is  ~ 60% larger than ours, and it requires four lumped capacitors and 12 vias to achieve a read distance of 10.2 m. The orientation-insensitive tag antenna in^15^ consists of two orthogonally placed dipolar patches that can be detected in all directions at any points above the backing metal. However, the tag’s read range is very low, and it has a poor impedance matching with a microchip. With the application of the characteristic mode, a dual-polarized UHF RFID tag antenna with polarization diversity feature has been proposed in^30^. However, the tag footprint is larger, while its read range is smaller than ours. Moreover, the tag is not flexible due to the use of FR4 rigid substrate. To achieve polarization diversity, the tag structure in^31^ consists of two folded crossed dipoles that are orthogonally displaced, where the read range is 50% lower than ours. It should be mentioned that all the tags in^9,15,30,31^ require the use of more than one radiator to enable accessibility in all polarizations. In contrast, our newly proposed tag here only needs a single radiator, and it can achieve good impedance matching and far read range at the same time. Our proposed tag is also compared with other compact metal-mountable tags^32,33^, although they are not polarization insensitive. A compact linearly polarized double-layered tag antenna^32^ is made by combining a dual-element planar inverted-F antenna (PIFA) fed by a capacitive network embedded inside the middle layer. Nevertheless, the fabrication process is extremely time-consuming due to the incorporation of four interlayer metallic vias, and the read range is much shorter as well. For metal-mountable applications, a compact C-shaped patch has been proposed in^33^; however, the antenna structure is made on a rigid substrate, and it is not flexible. The achievable read range is also not so far. Although the metal-mountable tags in^5,8,36^ are compact, their achievable read ranges are less than 7 m with single-polarization readability. The in-metal tag proposed by^34^ is too large, while the read distance for that in^35^ is too short. On the other hand, our newly presented tag antenna has a low profile and slight flexibility, and it can be applied on metal for achieving a far distance. Most importantly, it can be tactfully designed using a single radiator to achieve polarization insensitivity feature above the backing metal.

**Table 1:** Comparison with other metal tags (All are normalized with a reader power of 4 W EIRP).

Ref	Tag dimension (mm)	Application	Flexibility (substrate)	Polarization insensitivity	Read distance (m)
This work	40 × 40 × 3.38 (0.122λ × 0.122λ × 0.010λ)	On-metal	Yes (Foam)	Yes	15
^5^	20 × 18 × 1.7 (0.061λ × 0.055λ × 0.005λ)	On-metal	Yes (Foam)	No	6.62
^8^	18 × 4 × 2 (0.052λ × 0.011λ × 0.005λ)	On-metal	No (Ceramic Laminate)	No	3.32
^9^	64 × 64 × 2 (0.196λ × 0.196λ × 0.006λ)	On-metal	No (FR4)	Yes	10.2
^15^	30 × 30 × 1.6 (0.092λ × 0.092λ × 0.005λ)	On-metal	Yes (Foam)	Yes	3.5
^30^	50 × 50 × 2 (0.153λ × 0.153λ × 0.006λ)	On-metal	No (FR4)	Yes	8.5
^31^	40 × 40 × 1.6 (0.121λ × 0.121λ × 0.004λ)	On-metal	Yes (Foam)	Yes	7.7
^32^	26 × 14 × 2.4 (0.081λ × 0.043λ × 0.007λ)	On-metal	No (FR4)	No	5.5
^33^	30 × 30 × 3 (0.092λ × 0.092λ × 0.009λ)	On-metal	No (FR4)	No	6.4
^34^	117 × 80 × 3.18 (0.354λ × 0.242λ × 0.009λ)	In-metal	No (Rogers RT5880)	No	23
^35^	23 × 23 × 1 (0.071λ × 0.071λ × 0.003λ)	In-metal	No (Alumina)	No	0.6
^36^	23 × 7.5 × 3.2 (0.070λ × 0.023λ × 0.010λ)	On-metal	Yes (Foam)	No	5

## Conclusion

For the first time, in this paper, we have proposed a novel folded patch structure, which consists of two flaps of tightly overlapped patches on a single-layer resonator, for designing a polarization-insensitive tag antenna that can be used on metal. Here, the pair of orthogonal currents are generated in a unique way with the use of only one radiator. This approach is new as it does not require the use of several radiating components for designing a polarization-insensitive tag antenna. The tag antenna design here is rather simple, and the radiator is only composed by two flaps of patches, where they are partially overlapped for generating a pair of orthogonal currents. Such feature makes the tag readable from almost all directions at all points above the metal surface. To illustrate the design concept, simulations and measurements have been conducted. An equivalent circuit model was also built to explain the impedance and field characteristics. When tested on metal, the proposed tag was able to attain a maximum reading distance of ~ 15 m. The tag resonant frequency is also quite constant.

## Data Availability

The datasets used and/or analysed during the current study available from the corresponding author on reasonable request.
